# Campbell title registrations to date–February 2024, and discontinued protocols

**DOI:** 10.1002/cl2.1390

**Published:** 2024-02-28

Details of new titles for systematic reviews or evidence and gap maps that have been accepted by the Editor of a Campbell Coordinating Group are published in each issue of the journal. If you would like to receive a copy of the approved title registration form, please *send an email* to the Managing Editor of the relevant Coordinating Group.

A list of discontinued protocols appears below these new titles. If you are interested to continue a project, please get in touch with the Managing Editor of the relevant Coordinating Group or email info@campbellcollaboration.org.

## BUSINESS AND MANAGEMENT

Is the CEO–employee pay ratio related to subsequent firm performance in publicly traded companies? A systematic review

Denise Rousseau, Byeong Jo Kim, Cedric Velghe, Jangbum Lee, Ryan Splenda

16 January 2024

## CHILDREN AND YOUNG PERSONS WELLBEING

Exploring the relationship between breastfeeding and childhood pneumonia in sub‐Saharan Africa: A structured literature review

Faridat Sanusi, Claire Lewsey, Adeniyi Adeboye

17 January 2024

## CRIME AND JUSTICE

Risk and protective factors and interventions against child sexual abuse: An umbrella review

Izabela Zych, Inmaculada Marín‐López

10 February 2024

## DISABILITY

Protocol for a scoping review on stakeholder engagement in rehabilitation research for people with disabilities

Xinsheng Cindy Cai, Devin Dedrick

11 December 2023

The effectiveness of carer‐delivered rehabilitation programs on participation in daily activities and quality of life for children and adolescents with physical disabilities and motor‐based disorders: A systematic review and meta‐analysis

Nikki Tulliani, Caroline Mills, Lily Collison, Isabel Chapman, Paul Fahey, Karen P. Y. Liu

31 January 2024

## EDUCATION

The impact of integrated thematic instruction model on primary and secondary school pupils compared to standard teaching: A systematic review

Klára Barancová, Jiří Kantor, Zuzana Svobodova, Martina Fasnerová

5 December 2023

The effects of extending the school day on student achievement, behavior, and well‐being: A systematic review and meta‐analysis

Mikkel Vembye, Malene Kildemoes, Anne Nandrup, Elizabeth Bengtsen, Trine Filges

25 November 2023

## INTERNATIONAL DEVELOPMENT

Food environment, food choice, diets, and nutrition outcomes of pastoralists in Africa: Scoping review

Esther Omosa, Francoise Cattaneo, Matthew Kibbee, Paula Dominguez‐Salas, Natasha Bishop, Inge Brouwer

1 February 2024

Interventions to promote inclusive governance for underserved population in sub‐Saharan Africa: An evidence and gap map

David Sarfo Ameyaw, Takyiwaa Manuh, Clarice Panyin Nyan, Sheila Agyemang Oppong

26 January 2024

## SOCIAL WELFARE

Factors contributing to the discontinuation of breastfeeding upon women's return to work: A scoping review

Ana Scotta, Paula Barral, Ailin Farre, Elio Soria

5 February 2024

Mindfulness‐based interventions for improving tic‐related symptoms in children and adults with chronic tic disorder and Tourette's disorder: A systematic review

Ilana Singer, Méliza Gagnon, Julie Leclerc

11 December 2023

## DISCONTINUED PROTOCOLS

If you are interested to continue one of the projects below, please get in touch with the Managing Editor of the relevant Coordinating Group or email info@campbellcollaboration.org.

## CRIME AND JUSTICE

https://onlinelibrary.wiley.com/doi/10.1002/CL2.85

PROTOCOL: Mandatory arrest for misdemeanor domestic violence effects on repeat offending

Barak Ariel, Lawrence W. Sherman

## EDUCATION

https://onlinelibrary.wiley.com/doi/10.1002/CL2.163

PROTOCOL: Provision of information and communications technology (ICT) for improving academic achievement and school engagement in students aged 4–18

Kristin Liabo, Laurenz Langer, Antonia Simon, Kathy‐Ann Daniel‐Gittens, Alex Elwick, Janice Tripney

https://onlinelibrary.wiley.com/doi/10.1002/CL2.210

PROTOCOL: Universal school‐based programmes for improving social and emotional outcomes in children aged 3–11 years

Paul Connolly, Sarah Miller, Jennifer Hanratty, Jennifer Roberts, Seaneen Sloan

https://onlinelibrary.wiley.com/doi/10.1002/CL2.110

PROTOCOL: Education support services for improving school engagement and academic performance of children and adolescents with a chronic health condition

Michelle A. Tollit, Susan M. Sawyer, Savithiri Ratnapalan, Tony Barnett

https://onlinelibrary.wiley.com/doi/10.1002/CL2.197

PROTOCOL: Electronic mentoring to promote positive youth outcomes for young people under 25: A systematic review

Robyn M. O'Connor, David L. DuBois, Lucy Bowes

https://onlinelibrary.wiley.com/doi/10.1002/CL2.166

PROTOCOL: Merit pay programs for improving teacher retention, teacher satisfaction, and student achievement in primary and secondary education: A systematic review

Gary Ritter, Julie Trivitt, Leesa Foreman, Corey DeAngelis, George Denny

## INTERNATIONAL DEVELOPMENT

https://onlinelibrary.wiley.com/doi/10.1002/cl2.1122

PROTOCOL: Development evaluations in India 2000–2018: A country impact evaluation map

Ashrita Saran, Eti Rajwar, Bhumika T. V., Divya S. Patil, Howard White

https://onlinelibrary.wiley.com/doi/10.1002/cl2.1186

PROTOCOL: The association between whole‐grain dietary intake and noncommunicable diseases: A systematic review and meta‐analysis

Wasim A. Iqbal, Gavin B. Stewart, Abigail Smith, Linda Errington, Chris J. Seal

https://onlinelibrary.wiley.com/doi/10.1002/CL2.84

PROTOCOL: Nutrition interventions and programs for reducing mortality and morbidity in pregnant and lactating women and women of reproductive age: A systematic review

Philippa Middl

https://onlinelibrary.wiley.com/doi/10.1002/CL2.171

PROTOCOL: Agronomic biofortification strategies to increase grain zinc concentrations for improved nutritional quality of wheat, maize and rice: A systematic review

Israel F. N. Domingos, Marcin Baranski, Carlo Leifert, Ismail Cakmak, Zed Rengel, Paul E. Bilsborrow, Gavin B. Stewart

https://onlinelibrary.wiley.com/doi/10.1002/CL2.138

PROTOCOL: Improving maternal, newborn and women's reproductive health in crisis settings

Primus Che Chi, Henrik Urdal, Odidika U. J. Umeora, Johanne Sundby, Paul Spiegel, Declan Devane

https://onlinelibrary.wiley.com/doi/10.1002/CL2.183

PROTOCOL: The impacts of interventions for female economic empowerment at the community level on human development

Marcela Ibanez, Sarah Khan, Anna Minasyan, Soham Sahoo, Pooja Balasubramanian

https://onlinelibrary.wiley.com/doi/10.1002/CL2.154

PROTOCOL: Peer‐based interventions for reducing morbidity and mortality in HIV‐positive women

J. Petkovic, M. Doull, A. M. O'Connor, J. Aweya, M. Yoganathan, V. Welch, G. A. Wells, P. Tugwell

https://onlinelibrary.wiley.com/doi/10.1002/CL2.188

PROTOCOL: Community‐led total sanitation in rural areas of low‐ and middle‐income countries: A systematic review of evidence on effects and influencing factors

Josef Novotný, Jiří Hasman, Martin Lepič, Vít Bořil

https://onlinelibrary.wiley.com/doi/10.1002/CL2.140

PROTOCOL: Access to electricity for improving health, education and welfare in low‐ and middle‐income countries

Kavita Mathur, Sandy Oliver, Janice Tripney

https://onlinelibrary.wiley.com/doi/10.1002/CL2.157

PROTOCOL: Saving promotion interventions for improving saving behaviour and reducing poverty in low‐ and middle‐income countries

Janina Isabel Steinert, Ani Movsisyan, Juliane Zenker, Ute Filipiak, Yulia Shenderovich

https://onlinelibrary.wiley.com/doi/10.1002/CL2.172

PROTOCOL: The effect of interventions for women's empowerment on children's health and education

Sebastian Vollmer, Sarah Khan, Le Thi Ngoc Tu, Atika Pasha, Soham Sahoo

https://onlinelibrary.wiley.com/doi/10.1002/CL2.200

PROTOCOL: The effects of road infrastructure, and transport and logistics services interventions on women's participation in informal and formal labour markets in low‐ and middle‐income countries

Manisha Gupta, Souvik Bandyopadhyay, Meerambika Mahapatro, Shreya Jha

## SOCIAL WELFARE

https://onlinelibrary.wiley.com/doi/10.1002/cl2.1259

PROTOCOL: Effects of virtual reality exposure therapy versus in vivo exposure in treating social anxiety disorder in adults: A systematic review and meta‐analysis

Martin Polak, Norbert Tanzer, Per Carlbring

https://onlinelibrary.wiley.com/doi/10.1002/CL2.109

PROTOCOL: The therapeutic alliance and psychotherapy outcomes for young adults aged 18 to 34

Stacy J. Green, Julia H. Littell, Karianne Hammerstrøm, Emily Tanner‐Smith, Jessica Schaffner Wilen

https://onlinelibrary.wiley.com/doi/10.1002/CL2.56

PROTOCOL: Cognitive‐behavioural therapy for parents who have physically abused their children

Mogens N. Christoffersen, Jacqueline Corcoran, Diane DePanfilis, Claire Daining

